# Low Circulation of Subclade A1 Enterovirus D68 Strains in Senegal during 2014 North America Outbreak 

**DOI:** 10.3201/eid2507.181441

**Published:** 2019-07

**Authors:** Amary Fall, Mamadou Malado Jallow, Ousmane Kebe, Davy Evrard Kiori, Sara Sy, Déborah Goudiaby, Cheikh Saad Bouh Boye, Mbayame Ndiaye Niang, Ndongo Dia

**Affiliations:** Institute Pasteur de Dakar, Dakar, Senegal (A. Fall, M.M. Jallow, O. Kebe, D.E. Kiori, S. Sy, D. Goudiaby, M.N. Niang, N. Dia);; Aristide Le Dantec Teaching Hospital, Dakar (C.S.B. Boye)

**Keywords:** enterovirus, EV-D68, outbreak, ILI, ARI, SARI, respiratory infections, Senegal, surveillance, viruses

## Abstract

To retrospectively investigate enterovirus D68 circulation in Senegal during the 2014 US outbreak, we retrieved specimens from 708 persons, mostly children, who had acute respiratory symptoms during September–December 2014. Enterovirus D68 was detected in 14 children (2.1%); most cases occurred in October. Phylogenetic analysis revealed that all strains clustered within subclade A1.

A 2014 outbreak of ­>1,153 cases of respiratory enterovirus (EV) D68 infection in the United States was responsible for the deaths of 13 children (*1*). This occurrence led public health officials to improve surveillance systems worldwide. Enhanced surveillance revealed a continuous spread of EV-D68 in several North and South America countries, including Canada (*2*), Chile (*3*), and Mexico (*4*), and in several countries in Europe, including Germany (*5*), Denmark (*6*), and France (*7*). However, in Africa, data on the spread of EV-D68 from the 2014 US outbreak were scarce. This retrospective study sought to confirm the circulation of EV-D68 in Senegal during the 2014 outbreak and to characterize the molecular composition of any EV-D68 strains that circulated. 

## The Study 

For this retrospective case series, we studied records of children with acute respiratory illness (ARI) and, with consent, those of outpatients with influenza-like illness (ILI) attending sentinel sites dedicated to influenza surveillance, most recorded in Senegal during September–December 2014. For each patient, in addition to recording demographic and clinical data, healthcare personnel collected a nasopharyngeal swab specimen. As part of routine surveillance, specimens were initially tested for respiratory viruses including influenza, respiratory syncytial virus, adenoviruses, metapneumovirus, coronaviruses, parainfluenza, rhinoviruses, enteroviruses, and bocaviruses (*8*). Later, samples collected during September–December 2014 were screened for EV-D68, as described elsewhere (*9*). For the molecular studies, the viral capsid protein 1 (VP1) gene region of EV-D68 was amplified and sequenced as described elsewhere (*10*). 

Specimens were collected from 708 patients, ranging in age from 1 month to 95 years, and tested for EV-D68. The median age was 9 months; 45.8% of patients were children <5 years of age. The male-to-female ratio was 0.95. EV-D68 was detected in 14 patients (2.0%): 13 of 680 with ILI and 1 of 28 with ARI. A similar rate was reported by Poelman et al. (*11*) in a Europe-wide retrospective and prospective laboratory analysis of clinical specimens during July–December 2014. A single infection was found in 5 samples; 9 samples were found to have >1 additional respiratory virus (Table). EV-D68 detection was associated with cough in 12 of 14 patients and rhinitis in 8 of 14. The single patient with ARI who tested positive for EV-D68 was a 7-month-old child, with no underlying disease, who had clinical signs of acute bronchitis, including dry cough, pulmonary condensation, rhinorrhea, progressive breathing difficulty, and an unusually long duration of illness of >1 month; his condition deteriorated and he was hospitalized. This extended duration, linked to EV-D68 infection, was reported elsewhere (*12*). 

**Table:** Demographic, clinical characteristics and co-detections for the 14 patients infected with enterovirus D68, Senegal, September–December 2014*

NLABID	Illness type	Patient sex	Locality	Epidemiologic week	Clinical signs/symptoms	Co-detected infections
E1462/2014	ILI	F	Tambacounda	41	Fever, cough, rhinitis	AdV, HRV
E1464/2014	ILI	M	Dakar	41	Fever, cough, rhinitis, pharyngitis	AdV, EV
E1465/2014	ILI	F	Dakar	41	Fever, cough, rhinitis, pharyngitis	AdV
E1470/2014	ILI	M	Kaolack	41	Fever, cough, pharyngitis	AdV, EV, influenza B
E1486/2014	ILI	F	Mbour	41	Fever, cough, diarrhea	AdV, RSV
E1554/2014	ILI	M	Dielmo	42	Fever, rhinitis, myalgia	EV
E0048/2014	ARI	M	Dakar	42	Fever, cough, rhinitis	None
E1560/2014	ILI	F	Dielmo	42	Fever, cough, rhinitis	Influenza B, HRV
E1583/2014	ILI	M	Dakar	43	Fever, cough	EV, influenza B
E1626/2014	ILI	F	Fatick	43	Fever, cough, vomiting	AdV, PIV, influenza B
E1704/2014	ILI	F	Ziguinchor	44	Fever, cough	None
E1709/2014	ILI	M	Mbour	44	Fever, cough	None
E1787/2014	ILI	M	Dielmo	45	Fever, rhinitis	None
E1899/2014	ILI	F	Dakar	50	Fever, cough, rhinitis	None

*AdV, adenovirus; ARI, acute respiratory infection; EV, enterovirus; HRV, human rhinovirus; ILI, influenza-like illness; NLABID, laboratory identification number; PIV, parainfluenza virus; RSV, respiratory syncytial virus.

**Figure 1:**
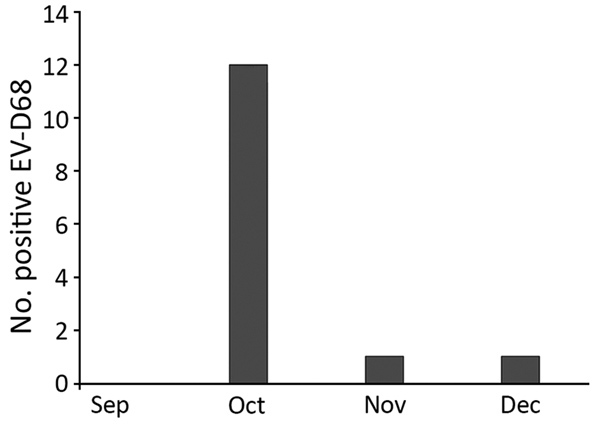
EV-D68 occurrence in Senegal, September–December 2014. A total of 708 nasopharyngeal samples were collected and tested for EV-68 during this period: 225 in September (0 positive), 218 in October (12 positive), 193 in November (1 positive), and 72 in December (1 positive). EV-D68, enterovirus D68.

The highest rate of infection during 2014 (46%) was recorded in the United States, probably at the beginning of the outbreak (*1*). In Senegal, most of the EV-D68–positive cases (12/14; 86.6%) were detected in October (Figure 1). Most EV-D68–positive patients (64.3%) were children <5 years of age. 

**Figure 2:**
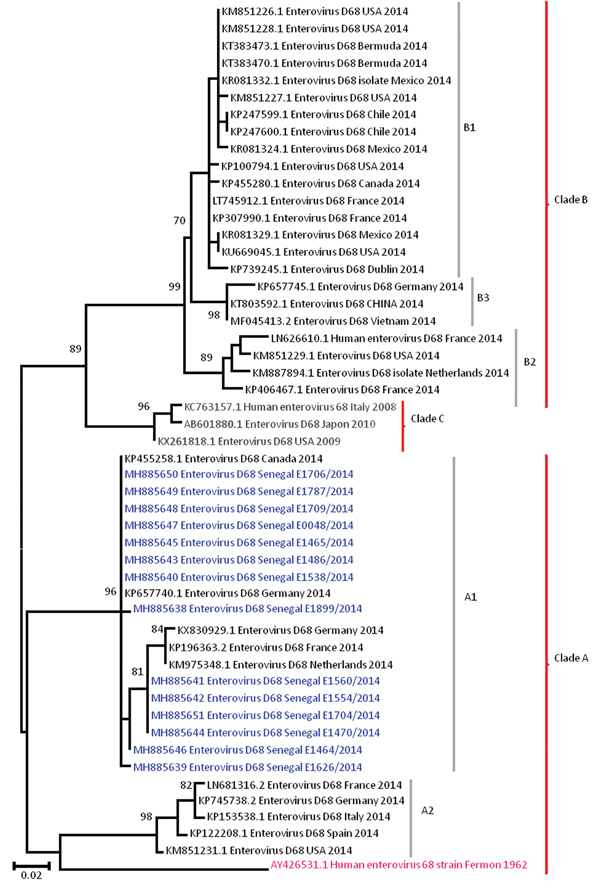
Phylogenetic relationships among enterovirus D68 (EV-D68) strains detected in Senegal (blue) and other countries (black) during the US outbreak period, September–December 2014. The phylogenetic tree based on nucleotide sequences of partial viral protein 1 genomic regions of EV-D68 strains was generated using the neighbor-joining method in MEGA6 (http://www.megasoftware.net). Sequences are identified by GenBank accession number, country, and period of detection. The phylogenetic tree is rooted by the oldest EV-D68 sequence in GenBank, the Fermon strain (pink), collected in 1962 in California, USA. We performed 1,000 bootstrap replicates to determine the consensus tree; support for nodes present in >70% of the trees are annotated. Scale bar indicates nucleotide substitutions per site.

From all 14 samples testing positive for EV-D68, we successfully obtained a 900-nt fragment of the VP1 gene; we deposited these sequences into GenBank (accession nos. MH885638–51). BLAST analysis (https://blast.ncbi.nlm.nih.gov/Blast.cgi) showed that all EV-D68 strains from Senegal shared >98% homology with strains detected in France (GenBank accession no. LN6813392), Canada (accession no. KP455258), and Germany (accession no. KP657740.1). Phylogenetic analysis of the VP1 fragment revealed that all 14 sequences belonged to the A1 variant of clade A (Figure 2). The strains from this study clustered with several other strains circulating during the same period in Germany, France, Philippines, Spain, Canada, the Netherlands, and Finland, with a bootstrap value of 97. However, genotype B, which had been detected in the United States, Canada, Germany, France, and other countries during the 2014 outbreak, was not found in Senegal. 

The phylogenetic evidence indicates EV-D68 circulation in Senegal, approximately concurrent with, but unrelated to, the large US outbreak in 2014. Results identified EV-D68 activity from October through December 2014 in Senegal, with peak EV-D68 detection occurring in October. This aligns with findings reported in countries in Europe (*11*); in North America, peaks were mapped in August in the United States (*13*) and in September in Canada (*14*).These results suggest circulation of the virus in Senegal 2 months after the beginning of the outbreak in the United States (*1*). This 2-month delay was also noted by Nathaniel et al. (*15*) before the introduction of EV-D68 strains in the Caribbean region. However, earlier EV-D68 circulation was reported in Latin America (*3*,*4*) and Europe (*5*–*7*). 

During the outbreak period, global circulation of EV-D68 strains belonging to clade B (B1 and B2, specifically) and strains from A1 and A2 subclades were reported (*1*,*7*,*11*). All EV-D68 strains identified in Senegal for this study belonged to subclade A1, unlike in the United States, where clade B EV-D68 strains circulated during almost the same period. Phylogenetically, the US outbreak was characterized by a novel subclade, B1, which emerged rapidly and was the dominant strain. Available data indicate that the exclusive circulation of clade A in Senegal during the 2014 outbreak was reported elsewhere only in Spain. In contrast, Slovenia, Norway (*11*), the Caribbean region (*15*), Mexico (*4*), and Chile (*3*) reported only clade B over the same period. 

Our study had some limitations. First, the inclusion of fever as a clinical sign for the case definition may have contributed to underestimating the number of EV-D68 infections during the North America outbreak; several studies have reported cases of afebrile EV-D68 infections (*13*). A second limitation was the small number of severe acute respiratory infection (SARI) cases in this study, because a higher proportion of detected EV-D68 in SARI patients or those with other concurrent conditions has been previously reported (*5*). At the time of data collection, the SARI sentinel surveillance system we used was not as developed as it is now. Inclusion in the screening of a larger number of hospitalized patients would probably present a more accurate picture of EV-D68 circulation. Finally, we conducted a retrospective study. The database we used contained limited information on disease outcome, and atypical clinical symptoms were not reported. Thus, the association between EV-D68 infections and severe clinical signs could not be established. 

## Conclusions

Despite the study’s shortcomings, we have confirmed circulation of EV-D68, exclusively of the A1 lineage, in Senegal at the time of the outbreak in the United States. Our study also adds to the growing body of evidence that EV-D68 may cause severe respiratory disease, especially in children, even in those without underlying chronic respiratory diseases. The detection of EV-D68 in a child with a severe respiratory infection reinforces the need to include this virus in SARI sentinel surveillance, which is focused mainly on pediatric hospitalization. Through this system, data on disease outcome, underlying complications, atypical clinical signs, duration of symptoms or hospitalization, and treatment are routinely collected to better assess burden. Additional research will be necessary to investigate cases of acute flaccid paralysis to determine their relationship with EV-D68 infections.

## References

[1] Midgley CM, Watson JT, Nix WA, Curns AT, Rogers SL, Brown BA, et al.; EV-D68 Working Group. Severe respiratory illness associated with a nationwide outbreak of enterovirus D68 in the USA (2014): a descriptive epidemiological investigation. Lancet Respir Med. 2015;3:879–87. 10.1016/S2213-2600(15)00335-5 26482320 PMC5693332

[2] Drews SJ, Simmonds K, Usman HR, Yee K, Fathima S, Tipples G, et al. Characterization of enterovirus activity, including that of enterovirus D68, in pediatric patients in Alberta, Canada, in 2014. J Clin Microbiol. 2015;53:1042–5. 10.1128/JCM.02982-14 25588657 PMC4390662

[3] Torres JP, Farfan MJ, Izquierdo G, Piemonte P, Henriquez J, O’Ryan ML. Enterovirus D68 infection, Chile, Spring 2014. Emerg Infect Dis. 2015;21:728–9. 10.3201/eid2104.141766 25811806 PMC4378481

[4] Vazquez-Perez JA, Ramirez-Gonzalez JE, Moreno-Valencia Y, Hernandez-Hernandez VA, Romero-Espinoza JA, Castillejos-Lopez M, et al. EV-D68 infection in children with asthma exacerbation and pneumonia in Mexico City during 2014 autumn. Influenza Other Respir Viruses. 2016;10:154–60. 10.1111/irv.12384 26935868 PMC4814865

[5] Reiche J, Böttcher S, Diedrich S, Buchholz U, Buda S, Haas W, et al. Low-level circulation of enterovirus D68-associated acute respiratory infections, Germany, 2014. Emerg Infect Dis. 2015;21:837–41. 10.3201/eid2105.141900 25898320 PMC4412236

[6] Midgley SE, Christiansen CB, Poulsen MW, Hansen CH, Fischer TK. Emergence of enterovirus D68 in Denmark, June 2014 to February 2015. Euro Surveill. 2015;20:21105. 10.2807/1560-7917.ES2015.20.17.21105 25955773

[7] Schuffenecker I, Mirand A, Josset L, Henquell C, Hecquet D, Pilorgé L, et al. Epidemiological and clinical characteristics of patients infected with enterovirus D68, France, July to December 2014. Euro Surveill. 2016;21:30226. 10.2807/1560-7917.ES.2016.21.19.30226 27195770

[8] Dia N, Diene Sarr F, Thiam D, Faye Sarr T, Espié E, OmarBa I, et al.; 4S Network Group. Influenza-like illnesses in Senegal: not only focus on influenza viruses. PLoS One. 2014;9:e93227. 10.1371/journal.pone.0093227 24675982 PMC3968133

[9] Tokarz R, Firth C, Madhi SA, Howie SR, Wu W, Sall AA, et al. Worldwide emergence of multiple clades of enterovirus 68. J Gen Virol. 2012;93:1952–8. 10.1099/vir.0.043935-0 22694903 PMC3542132

[10] Piralla A, Girello A, Premoli M, Baldanti F. A new real-time reverse transcription-PCR assay for detection of human enterovirus 68 in respiratory samples. J Clin Microbiol. 2015;53:1725–6. 10.1128/JCM.03691-14 25694533 PMC4400749

[11] Poelman R, Schuffenecker I, Van Leer-Buter C, Josset L, Niesters HG, Lina B; ESCV-ECDC EV-D68 study group. European surveillance for enterovirus D68 during the emerging North-American outbreak in 2014. J Clin Virol. 2015;71:1–9. 10.1016/j.jcv.2015.07.296 26364237

[12] Imamura T, Oshitani H. Global reemergence of enterovirus D68 as an important pathogen for acute respiratory infections. Rev Med Virol. 2015;25:102–14. 10.1002/rmv.1820 25471236 PMC4407910

[13] Oermann CM, Schuster JE, Conners GP, Newland JG, Selvarangan R, Jackson MA. Enterovirus d68. A focused review and clinical highlights from the 2014 U.S. outbreak. Ann Am Thorac Soc. 2015;12:775–81. 10.1513/AnnalsATS.201412-592FR 25714788

[14] Peci A, Winter AL, Warshawsky B, Booth TF, Eshaghi A, Li A, et al. Epidemiology of enterovirus D68 in Ontario. PLoS One. 2015;10:e0142841. 10.1371/journal.pone.0142841 26599365 PMC4658075

[15] Nathaniel S, Ahmed S, Wilson J, Gutierrez C, Chadee DD, Olowokure B, et al. First reported enterovirus D68 infection in pediatric patients from the Caribbean region: evidence of spread from the U.S. outbreak. Rev Panam Salud Publica. 2017;41:e11. 28443999 10.26633/RPSP.2017.11 PMC6660861

